# Can *Anganwadi* services strengthening improve the association between maternal and child dietary diversity? Evidence from Project Spotlight implemented in tribal dominated Gadchiroli and Chandrapur districts of Maharashtra, India

**DOI:** 10.1371/journal.pone.0264567

**Published:** 2022-03-03

**Authors:** Abhishek Kumar, Sunil Rajpal, Ruby Alambusha, Smriti Sharma, William Joe

**Affiliations:** 1 Centre for Studies in Economics and Planning, Central University of Gujarat, Gujarat, India; 2 School of Liberal Education, FLAME University, Lavale, Maharashtra, India; 3 Population Research Centre, Institute of Economic Growth, Delhi University Enclave (North Campus), Delhi, India; 4 Tata Trusts, R. K. Khanna Tennis Stadium, Africa Avenue, New Delhi, India; ESIC Medical College & PGIMSR, INDIA

## Abstract

Dietary intake is a fundamental determinant of maternal and child nutrition. This paper presents evidence on whether maternal and child dietary diversity can be improved with systemic improvements focused on strengthening training, capacity building, and behavior change communication among frontline workers to encourage improved nutritional practices among mothers and children in the intervention area. The evidence is derived from Project Spotlight intervention that was jointly implemented by Department of Women and Child Development, Government of Maharashtra and Tata Trusts in tribal dominated Gadchiroli and Chandrapur districts in Maharashtra. Based on a pre-post comparison of baseline (2019) and endline (2021) household survey data it is confirmed that there is a significant association between maternal and child dietary diversity in the study area. Notably, dietary diversity in mother-child dyads is marked with a higher consumption of fruits and vegetables as well as eggs and flesh foods. Econometric analysis further reveals that the association between maternal and child dietary diversity has improved after the systems strengthening interventions. The paper concludes that local interventions such as Project Spotlight for strengthening counselling services and coverage by frontline workers and enhancing knowledge and awareness on maternal and child dietary diversity among communities are important for improving maternal and child nutrition.

## Introduction

Dietary intake and behaviors are a critical determinant of maternal and child nutrition. Poor and inadequately diversified diet can impair cognitive and physical development among children and can render long term consequences even during adolescence and adulthood [1–4]. Dietary deprivations increase susceptibility to life-threatening infections and are associated with an elevated risk of maternal and child mortality [5–7]. Despite such relevance, there is limited research and policy engagements with assessments of maternal and child dietary intake practices and interventions. This stands in stark contrast with the case of anthropometric indicators which has received disproportionate focus and attention.

The World Health Organization recommends that a child must consume food from at least 4 groups including at least one fruit or vegetable, one animal-source food along with other staple food of the region [8]. The infant and young child feeding (IYCF) guidelines also emphasize on consumption from 4 or more groups for children 6–23 months who are on breastfeeding. This can also help correct micronutrient deficiency among children [9]. A regular and diversified diet is also a marker of quality of diet among adults and is a proxy indicator to facilitate a rapid assessment of nutrient intake than complex dietetics details [10–12].

Given such relevance, an assessment of IYCF practices across high-risk geographies offers insights on maternal and child nutrition and can be helpful to develop local interventions [8]. In this respect, nutrition counselling and behavior change communication based interventions have shown good results in improving maternal and child dietary diversity [5]. Such assessments, however, should also focus on aspects of maternal diet which are strongly associated with dietary diversity among children [13–17]. In particular, it is equally important to understand whether such interventions can further boost the association between maternal and child diets and dietary diversity.

With this motivation, we aim to present evidence on whether local interventions focusing on strengthening of nutrition related services and delivered via the network of frontline workers can improve and strengthen the association between maternal and child dietary diversity. Specifically, the analysis focuses on the experience of Project Spotlight in tribal dominated district of Gadchiroli and Chandrapur jointly implemented by the Department of Women and Child Development (DWCD), Government of Maharashtra and the Tata Trusts. Project Spotlight has focused on systems strengthening and community mobilization activities in these two districts. These activities directly support the *Anganwadi* center (AWC, courtyard shelter) services delivered across rural and urban areas under the flagship Integrated Child Development Services (ICDS) of India.

The ICDS delivers six specific *Anganwadi* services with the help of *Anganwadi* workers (AWW). This includes provisioning of supplementary nutrition and health and nutrition education for pregnant and lactating mothers as well as children (6 months to 6 years). The AWWs are responsible for enhancing knowledge and awareness on maternal and child diets that can lead to improvements in dietary diversity and practices. However, several studies have noted that AWWs lack training and capacity building and perceive this as a barrier in improving service delivery [18]. In this context, Project Spotlight assumes significance because of its focus on training, capacity building, and behavior change communication to adopt improved nutritional practices in the intervention area. The system strengthening initiatives under the project also supported refurbishment of AWCs and widespread community-based events (*Jan Andolan*) as envisaged under the POSHAN *Abhiyaan* to promote knowledge and awareness on diet and nutrition.

This paper examines the baseline (2019) and endline (2021) information of the project to comprehend the association between maternal and child dietary diversity in the intervention areas (Chandrapur and Gadchiroli districts of Maharashtra) which have received training for capacity building of the workers of the AWCs. It also examines the improvements in the association after the intervention phase. The four specific objectives are as follows: (a) to assess the consumption of various food groups and overall maternal and child dietary diversity levels; (b) to identify the food groups which are less likely to be consumed by mother-child dyads; (c) to examine the changes in the association between maternal and child dietary diversity after the project activities; and (d) to estimate the potential dietary diversity that can be achieved with consumption of certain selected food groups. The findings will be important because it can demonstrate that the association between maternal and child diets can be strengthened through local interventions focusing on training and capacity building of frontline workers on behavior change communication and nutrition counselling.

## Data and methods

The analysis is based on the household survey data collected by The India Nutrition Initiative (TINI) with ethical approval from the SIGMA Institutional Review Board. The households were selected based on a systematic random sample design. Two cross-sectional survey were conducted in 2019 and 2021. The survey in 2019 (baseline) covered a sample of 460 children in 0–23 age bracket while the survey in 2021 (endline) covered 450 children (2021) across three administrative blocks in Chandrapur and 2 administrative blocks in Gadchiroli. For the survey purposes, AWCs were selected based on a stratified random sample with blocks serving as unit for stratification. These AWCs were focused through various forms of support such as training and capacity building of the frontline workers managing the AWCs, through infrastructure up-gradation of the AWCs and improving community level knowledge and awareness through various events and interactions. The list of households (with children below 6 years of age) was obtained from the AWC registers and the respondents were randomly selected from the list. The final analytical sample provides diet-related information for 692 mother-child pairs with children aged 6–23 months.

Information on 16 food items (S1 Table), that were consumed by child a day before was categorized into 7 groups as follows: a) dairy; b) grain, white roots and tubers; c) other fruits and vegetables; d) vitamin A rich fruits and vegetables; e) eggs; f) flesh food; g) pulses, nuts and seeds. Dietary diversity is defined as consumption of food from at least 4 food groups following the WHO IYCF guidelines [10] and is coded as “1” if the child is consuming a diversified diet and “0” otherwise. Information on 10 food groups was collected from mothers. These include: a) grains, white roots and tubers; b) pulses; c) nuts and seeds; d) dairy; e) meat, poultry, fish; f) eggs; g) dark green leafy vegetables; h) vitamin A rich fruits and vegetables; i) other fruits and vegetables; j) beans, peas and lentils. Diversified diversity (DD) is defined as consumption from at least 5 food groups based on review of literature [19] and is coded as “1” and “0” otherwise.

The selection of the socio-economic variables and their categorization is based on a thorough literature review [30]. The demographic correlates of mother and the child are included in the analysis with focus on maternal age, maternal education, social category, child’s birth weight and birth order. Maternal age was coded in three categories viz. a) less than 24 years, b) 25 to 29 years and c) more than 30 years. Maternal education variable was dichotomized as those with below primary and those with above primary education. Sex of child categorized as male and female as well as an indicator for low birth weight (yes/no) were also included. Household poverty status based on self-assessment includes two categories (poor and middle/rich). The categories of caste are based on the administrative classification adopted by Government of India and include: Schedule Caste (SC), Schedule Tribe (ST), Other Backward caste (OBC) and Others which contains all those who do not belong to SC, ST or OBC.

Descriptive statistical analysis is used to examine the dietary diversity patterns and consumption from different food groups by mother and the child. Based on the definition of consumption from 5 or more food groups, dietary diversity score is computed for the respondents. The dietary diversity score for child is computed on consumption from 4 or more food groups. The association of diversified dietary intake with the socio-economic characteristics is examined using simple linear regression, logistic regression as well as multilevel logistic regression models with diversified diet as the dependent variable and other socioeconomic variables as the key explanatory variables. Our data has a two-level hierarchical structure with children (level1) nested within administrative blocks.

Based on the regression model, we also estimated discriminatory accuracy to examine the concordance between maternal and child dietary diversity. For this purpose, the area under receiver operating characteristic curve (AUC) is estimated to quantify the accuracy of maternal dietary diversity information alone for identifying children with dietary diversity [20–21]. The AUC reflects the ability to classify between true and false cases of concordance based on the binary classification. An AUC value of 1 suggests that the model is able to perfectly distinguish between all the concordant and discordant relationships. On the contrary, an AUC value of 0 will indicate that all concordant relationships are predicted as discordant and vice versa. Typically, an AUC value of greater than 0.5 is an indication that the model is able to perform better in classifying the concordant relationships. Higher value of AUC reflects greater ability to identify such concordance. The AUC values presented here are based on post-estimates from both simple and multilevel logistic regression analysis where child dietary diversity is the outcome variable and maternal dietary diversity is the key independent variable. The AUC estimates are presented for both bivariate case as well as models adjusted for demographic and socioeconomic variables.

Finally, we use the population attributable risk (PAR) estimation framework to examine the potential dietary diversity (PDD) achievable through focus on universal inclusion of certain selected food groups among children [22]. The PDD values are derived based on post-estimates from the logistic regression model whereby the increase in dietary diversity score is computed for selected food groups based on the relative odds as observed in the sample. The following formulae is used for estimating PDD:

$$PDD=\frac{(RR-1)*P_{e}}{(RR-1)*P_{e}+1}$$

Where, RR is relative risks (odds), P_e_ is the prevalence of the food group of interest and PDD is potential dietary diversity. The estimations were carried out using Stata package *regpar* [25]. The estimated PDD value can be interpreted as the percentage increase in child dietary diversity that would be observed if there is a cent per cent consumption from a selected food group with other factors remaining unchanged. For instance, a PDD value of 10 percent for eggs would indicate that, with other factors held constant, universal consumption of eggs would increase the dietary diversity by 10 percentage points. All the analysis is performed using Stata 15.0.

### Ethics statement

Ethics committee approval for the study was received from SIGMA Institutional Review Board (New Delhi) in accordance with the compliance of Title 45, Code of Federal Regulations, subpart A (Common Rule) of NIH. Also, the respondents were informed about the survey and a verbal consent was taken prior to the interview.

## Results

The intervention is placed in a low-income setting (S1 Table). Considerable proportion of the mothers in the area (with child aged 6–23 months) has not completed primary education (40% in 2019 and 37% in 2021). Most of them identify or perceive themselves as poor households (54% in 2019 and 43% in 2021). Almost one-half of the sample respondents belonged to the scheduled tribes (ST) community. Given the socioeconomic profile of the region, direct interventions for maternal and child nutrition and counselling can be critical to improve dietary practices in the region.

Tables 1 and 2 presents the consumption patterns of children 6–23 months from the seven food groups. A high proportion of children consume grains and roots (88% in 2019 and 85% in 2021) followed by dairy (69% in 2019 and 62% in 2021). Consumption of eggs among children increased from 33% to 56% and of vitamin A rich fruits and vegetables increased from 33% to 62% between 2019 and 2021. Table 2 presents the food groups consumed by mothers. Most of the mothers reported consumption from food groups such as grains, roots and tubers as well as pulses, nuts and seeds. Consumption of dairy products and eggs has increased from 14% to 24% and from 14% to 36%, respectively.

**Table 1:** Dietary intake from food groups among children (6–23 months), Gadchiroli and Chandrapur, 2019 and 2021.

	Children (6–23 months)			
	2019		2021	
	N	%	N	%
Grains, white roots and tubers	274	87.5	321	84.7
Pulses, nuts and seeds	65	20.8	85	22.4
Dairy	215	68.7	234	61.7
Meat, poultry and fish	29	9.3	51	13.5
Eggs	105	33.5	211	55.7
Vitamin A rich fruits and vegetables	105	33.5	235	62.0
Other fruits and vegetables	152	48.6	201	53.0

**Table 2:** Dietary intake from food groups among mothers of children (6–23 months), Gadchiroli and Chandrapur, 2019 and 2021.

	Mothers (of children 6–23 months)			
	2019		2021	
	N	%	N	%
Grains, White Roots and Tubers	311	99.4	373	98.4
Pulses	263	84	324	85.5
Nuts and Seeds	24	7.7	49	12.9
Dairy	45	14.4	93	24.5
Meat, Poultry, Fish	68	21.7	70	18.5
Eggs	44	14.1	135	35.6
Dark green leafy vegetables	100	31.9	133	35.1
Other Vit A rich fruits and vegetables	16	5.1	29	7.7
Other vegetables	216	69	322	85
Other fruits	54	17.3	82	21.6

Dietary diversity among mothers and children is estimated to be much lower (Table 3). Altogether, 36% children (6–23 months) in 2019 and 54% in 2021 have adequate dietary diversity. Girls have higher dietary diversity than boys in both 2019 (41%) and 2021 (56%). In baseline and endline assessment, a higher percentage (34% and 45%, respectively) of children of low birth weight is consuming a less diversified diet. Further, Table 3 also presents maternal dietary diversity. Altogether, 20% mothers in 2019 and 37% in 2021 have adequate dietary diversity. Children with mothers consuming a diversified diet have higher dietary diversity (46% in 2019 and 60% in 2021.) than those who have not. Besides, respondents belonging to middle-rich category also have higher maternal dietary diversity than those who are poor. Dietary diversity is higher among mothers in the younger age group of 25–29 years.

**Table 3:** Dietary diversity among mothers and children (6–23 months) by background characteristics, Gadchiroli and Chandrapur, 2019 and 2021.

Background characteristics	Children (6–23 months)				Mothers (of children 6–23 months)			
	2019		2021		2019		2021	
	N	%	N	%	N	%	N	%
Maternal education								
Up to Primary	44	34.9	67	48.2	14	11.1	40	28.8
Above Primary	70	37.4	139	57.9	47	25.1	101	42.1
Maternal age								
15–24 years	47	31.1	94	53.4	20	13.2	50	28.4
25–29 years	51	39.8	91	54.8	34	26.6	74	44.6
30 years and above	16	47.1	20	55.6	7	20.6	16	44.4
Social group								
Scheduled castes	11	29.7	23	47.9	6	16.2	15	31.3
Scheduled tribes	54	35.5	90	50.6	21	13.8	59	33.1
Other backward classes	33	41.3	66	64.1	20	25	53	51.5
Others	16	37.2	25	54.3	13	30.2	12	26.1
Sex of the child								
Female	65	41.4	105	56.1	31	19.7	65	34.8
Male	49	31.4	93	53.4	30	19.2	66	37.9
Low birthweight of child								
No	82	37.6	147	59.0	47	21.6	102	41
Yes	32	33.7	59	45.4	14	14.7	39	30
Self-reported economic status								
Poor	52	31.3	91	57.2	21	12.7	51	32.1
Middle class or rich	61	43.0	110	51.4	39	27.5	88	41.1
Maternal dietary diversity								
No	55	29.6	54	42.5				
Yes	59	46.5	152	60.3				
All	114	36.4	206	54.4	61	19.5	141	37.2

**Fig 1:**
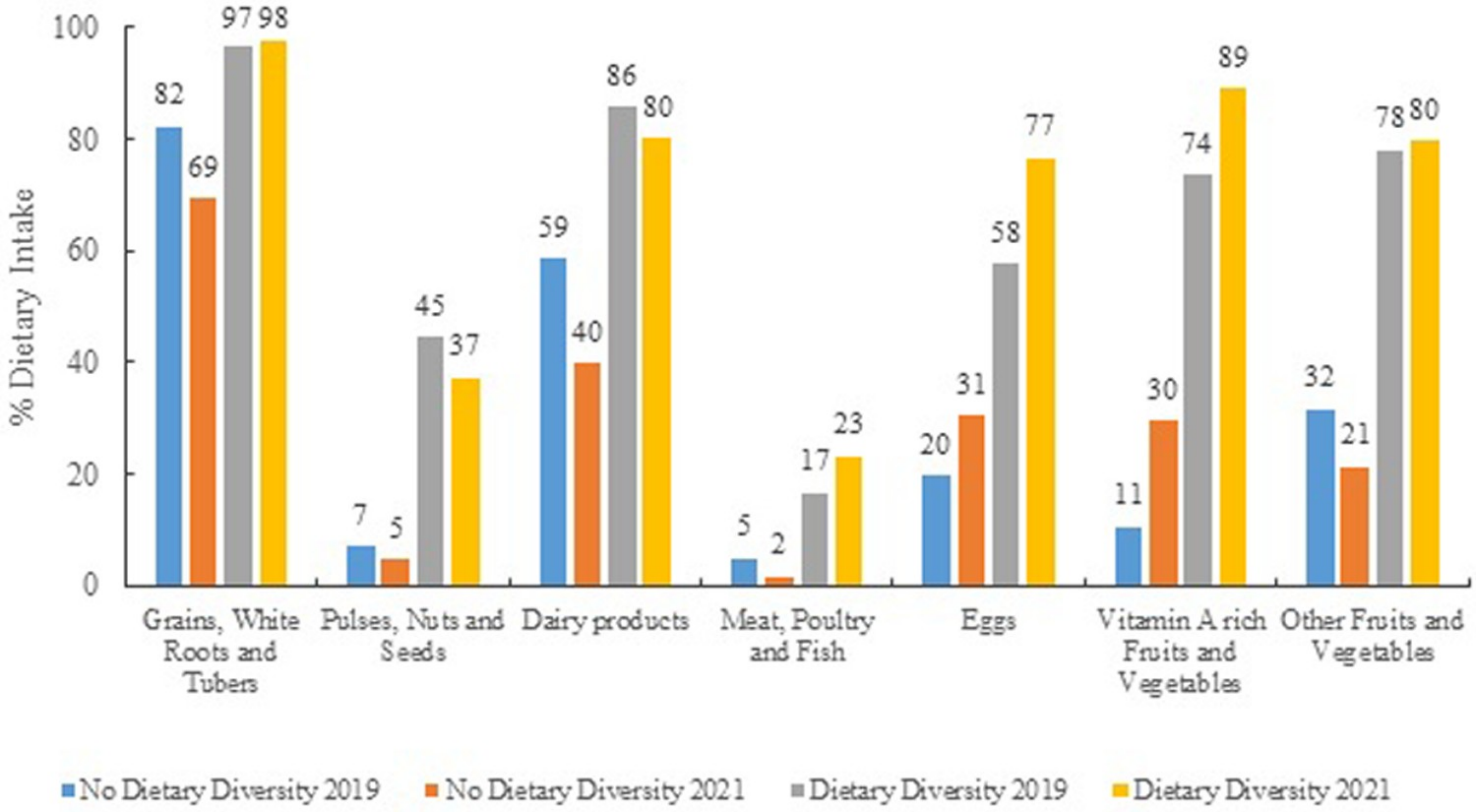
Dietary intake among children (6–23 months) across food groups by their dietary diversity status, Gadchiroli and Chandrapur, 2019 and 2021.

Fig 1 shows the consumption from different food groups based on the dietary diversity status of the child. Among children with dietary diversity higher consumption of eggs, dairy products, vitamin A rich fruits and vegetables, pulse, nuts and seeds as well as flesh foods is noted. For instance, in 2019, more than 75% children with dietary diversity consume eggs whereas only 31% children consume eggs if they do not have adequate dietary diversity. Similarly, 80% children with dietary diversity consume dairy products whereas it is only 40% for others. Similarly, S1 Fig shows that maternal dietary diversity can be also associated with a higher intake of dairy products, eggs, nuts and seeds, dark green leafy vegetables, fruits and vegetables including vitamin A rich fruits and vegetables. In 2021, 50% of mothers with adequate dietary diversity reported consumption of eggs whereas only 27% without dietary diversity consume eggs. Mothers who lack a diversified diet predominantly consume grains, roots, or tubers and lentils. The trends and patterns are consistent across during both baseline and endline assessment.

**Fig 2:**
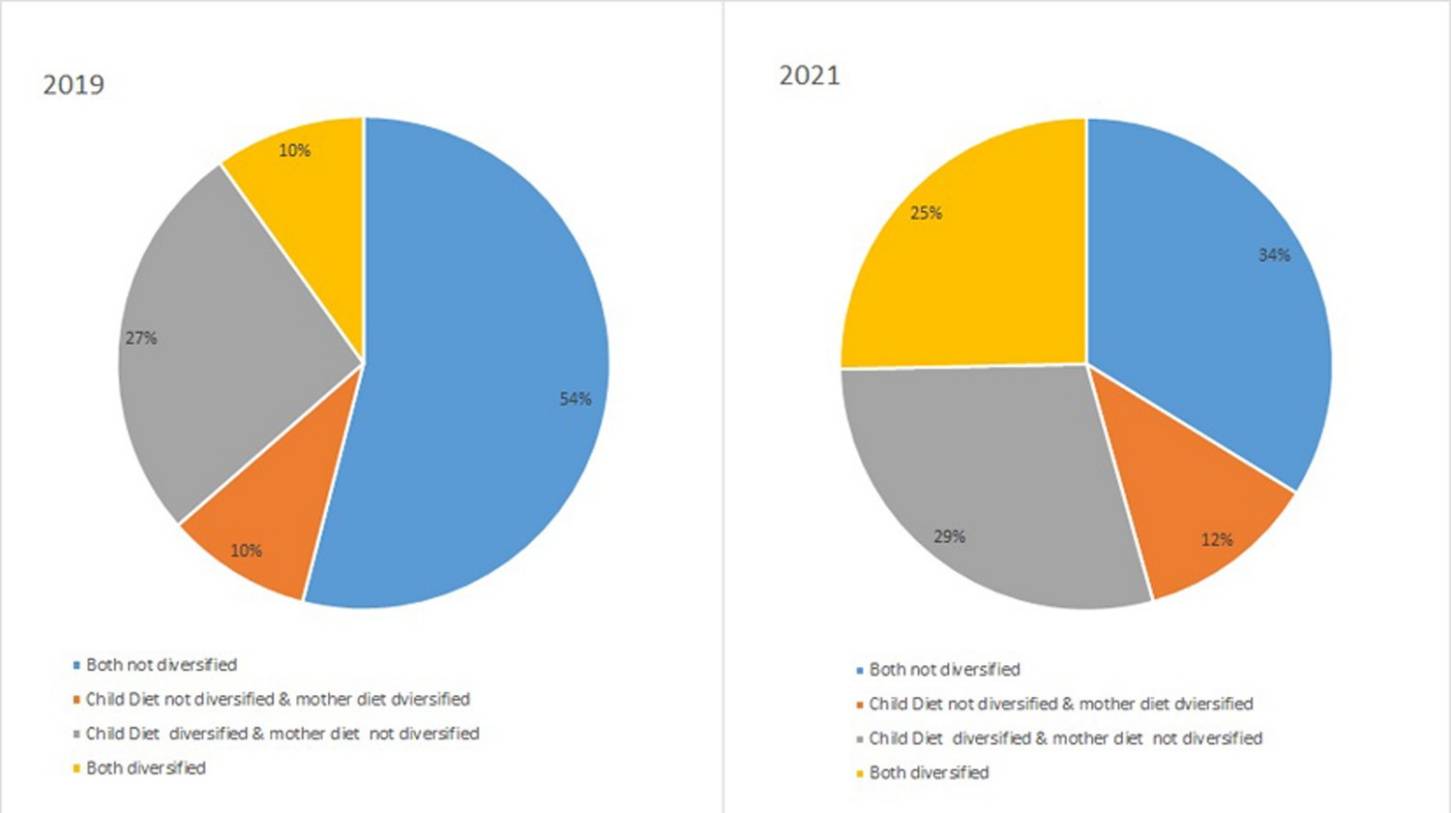
Dietary diversity status among mother-child dyads, Gadchiroli and Chandrapur, 2019 and 2021.

**Fig 3:**
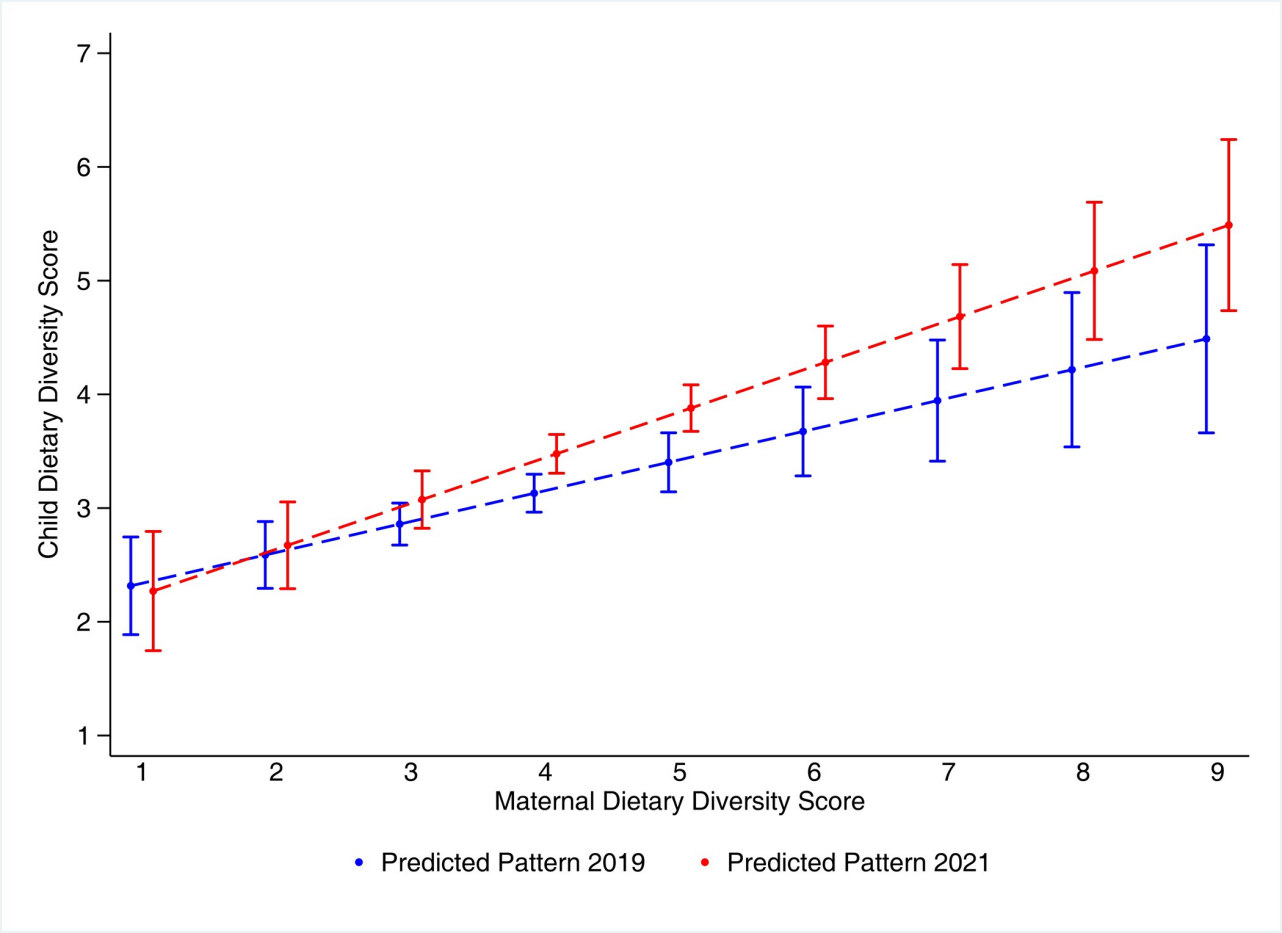
Association between number of food group intake among mothers and children (6–23 months), Gadchiroli and Chandrapur, 2019 and 2021.

Fig 2 shows that in 2019, 10% of the mother-child pairs had a diversified diet whereas in 54% cases both lacked dietary diversity. In 10% cases mother alone was found to consume a diversified diet whereas in 27% cases the child alone was found to have diverse diet. The situation has improved in 2021 with 25% mother-child pairs showing dietary diversity whereas the proportion lacking dietary diversity among both mother and child had reduced to 34%. S3 Table shows that dietary diversity among mother-child dyads, particularly during endline survey, was driven by higher consumption of vegetables and fruits as well as intake of animal flesh foods and eggs. Fig 3 further confirms this association between maternal and child dietary diversity. It is noted that higher numbers of food group consumption among mothers is positively associated with food group consumption among children. The predicted marginal effect on child dietary diversity from the linear regression model on food group consumption by mothers has a positive slope (0.27 for 2019 and 0.40 for 2021). This suggests that the association between maternal and child dietary diversity has strengthened between the baseline and endline assessments.

Results from both simple and multilevel logistic regression analysis confirm the association between maternal and child dietary diversity (Table 4 and S3 Table). In 2019, in the unadjusted models 1 (logistic) and 5 (multilevel logistic), maternal dietary diversity is associated with 2.10 times (OR 2.10; 95% CI [1.19, 3.71]) higher chances of child dietary diversity. After adjusting for the demographic and socioeconomic variables, the effect is noted to be similar and significant (OR 2.01; 95% CI [1.08, 3.74]) in the multilevel logistic regression model 6 for baseline and model 8 for end line (OR 2.26; 95% CI [1.40,3.65]). The concordance between maternal and child dietary diversity is further confirmed by the AUC estimates. The AUC values range from 0.56 in model 1 to 0.67 in model 8. All the values are greater than 0.5 and thus suggest high concordance between maternal and child dietary diversity. The strength of the association is much prominent in case of the adjusted multilevel logistic model 8 estimated on end line data (AUC = 0.67).

**Table 4:** Logistic regression based odds ratio and AUC coefficient for the association between maternal and child dietary diversity, Gadchiroli and Chandrapur, 2019 and 2021.

Background variables	Simple Logistic				Multilevel Logistic			
	2019	2019	2021	2021	2019	2019	2021	2021
	Model-1	Model-2	Model-3	Model-4	Model-5	Model-6	Model-7	Model-8
Maternal diet diversified	Unadjusted	Adjusted	Unadjusted	Adjusted	Unadjusted	Adjusted	Unadjusted	Adjusted
No	1	1	1	1	1	1	1	1
Yes	2.10*	2.03*	2.48***	2.26***	2.10*	2.01*	2.48***	2.26***
95% CI	[1.19,3.71]	[1.10,3.77]	[1.60,3.84]	[1.40,3.65]	[1.19,3.71]	[1.08,3.74]	[1.60,3.84]	[1.40,3.65]
AUC	0.56	0.65	0.60	0.67	0.56	0.66	0.60	0.67
95% CI	[0.51,0.61]	[0.59,0.71]	[0.56,0.65]	[0.62,0.73]	[0.51,0.61]	[0.6,0.72]	[0.56,0.65]	[0.62,0.73]
N	313	307	379	351	313	307	379	351

Note: The models are adjusted for demographic and socioeconomic characteristics of mother and children (S4 Table).

Table 5 shows that the odds of child dietary diversity have improved between 2019 and 2021.

Both unadjusted and adjusted models (model 1 and 2, respectively) show that compared to 2019, the children are 1.8 to 1.9 times more likely to have dietary diversity in 2021.

**Table 5:** Logistic regression based odds ratio for the association between maternal and child dietary diversity and role of Project Spotlight, Gadchiroli and Chandrapur, 2019 and 2021.

	Model-1	Model-2	Model-3
Maternal dietary diversity			
No	1	1	-
Yes	2.33***	2.14***	-
	[1.65,3.30]	[1.48,3.10]	
Year			
2019	1	1	-
2021	1.83***	1.90***	-
	[1.33,2.50]	[1.37,2.63]	
MDD Status x Year			
No MDD x 2019	-	-	1
No MDD x 2021	-	-	1.87**
			[1.28,2.73]
MDD x 2019	-	-	2.06*
			[1.14,3.74]
MDD x 2021	-	-	4.09***
			[2.56,6.54]
Maternal education			
Up to Primary	-	1	1
Above Primary	-	1.15	1.16
		[0.81,1.64]	[0.81,1.64]
Maternal age	-	1	1
25–29 years	-	1.06	1.06
		[0.75,1.50]	[0.75,1.50]
30 years and above	-	1.44	1.44
		[0.82,2.51]	[0.82,2.51]
Social group			
Scheduled castes	-	1	1
Scheduled tribes	-	1.23	1.23
		[0.74,2.07]	[0.73,2.07]
OBC	-	1.65	1.65
		[0.94,2.90]	[0.94,2.89]
Other	-	1.39	1.39
		[0.74,2.62]	[0.74,2.63]
Sex of the child			
Female	-	1	1
Male	-	0.77	0.77
		[0.56,1.06]	[0.56,1.06]
Low birth weight of child			
No	-	1	1
Yes	-	0.71*	0.71*
		[0.50,1.00]	[0.50,1.00]
Self-reported economic status			
Poor	-	1	1
Middle-Rich	-	0.87	0.88
		[0.62,1.23]	[0.62,1.23]
N	692	658	658

Further, model 3 shows that the association between maternal and child dietary diversity has also strengthened. Children with adequate maternal dietary diversity in 2019 were 2 times more likely (OR 2.06; 95% CI [1.14, 3.74]) to have a diverse diet than those with no diversity. However, in 2021, the dietary diversity is likely to be 3.6 times higher among children with adequate maternal dietary diversity than the reference group (OR 4.09; 95% CI [2.56,6.54]). As sensitivity analysis, we also ran separate regressions and noted similar improvements and strengthening of association in both Chandrapur and Gadchiroli districts (S4 Table).

Finally, we apply the population attributable risk (PAR) framework to present estimates on potential dietary diversity among children if universal coverage of certain selected food groups. Table 6 shows that universal and daily provisioning of eggs can increase dietary diversity from a base level of 54% to 68% thus yielding a net increase of 14 percentage point in child dietary diversity score. Universal consumption of flesh foods can increase dietary diversity by 27 percentage point (from 54% to 82%). Focus on beans, peas and lentils as well as inclusion of vitamin A rich fruits and vegetables can also lead to 16 percentage point increment in level of child dietary diversity.

**Table 6:** PAR analysis based potential dietary diversity (%) achievable among children (6–23 months) through planning a universal coverage of selected food groups, Gadchiroli and Chandrapur, 2019 and 2021.

Food groups	Child Dietary Diversity, 2019			Child Dietary Diversity, 2021		
	Base	Potential	Change	Base	Potential	Change
Eggs	0.37	0.54	-0.17	0.54	0.68	-0.13
Flesh food	0.37	0.55	-0.18	0.54	0.81	-0.27
Beans, peas or lentils	0.37	0.69	-0.32	0.54	0.79	-0.25
Vitamin A rich fruits and vegetables	0.37	0.69	-0.33	0.54	0.70	-0.15

## Discussion and conclusion

A direct focus on enhancing maternal and child dietary diversity is critical to ensure sustained improvements in maternal and child undernutrition. This requires local level interventions to improve knowledge and awareness through interpersonal as well as group counseling on importance of diet for well-being of both mother and the child. We present evidence from Project spotlight to suggest that maternal and child dietary diversity is related and this association can be strengthened through interventions that aim to strengthen existing systems and improve capacities of frontline workers on dietary counseling. The analysis puts forth the following salient findings. First, the levels of dietary diversity among mothers are lower than the levels among children. This is a clear opportunity to improve both maternal and child dietary diversity by encouraging food consumption across all groups that is usually consumed within a household. Second, there is a significant association between maternal and child dietary diversity in the study area. Econometric analysis as well as concordance analysis based on the AUC framework confirms the association. Third, the association between maternal and child dietary diversity has improved after the systems strengthening activities under project spotlight. Notably, these activities had a direct focus on training and capacity building of the frontline workers (AWWs) for improving dietary counseling in the community. Fourth, dietary diversity in mother-child dyads is marked with a higher consumption of fruits and vegetables as well as eggs and flesh foods. If some of these food groups are made available to all through interventions then it can lead to higher potential change in dietary diversity levels in the community.

The study findings, however, are not free of certain limitations. The information related to dietary intake from various food groups is based on a 24 hour dietary recall of consumption of any such item. But it does not necessarily capture the dietary content in terms of quantity and nutritional make up of the overall intake. This is an important aspect as minimum dietary diversity should also be accompanied with a minimum dietary frequency and quantity. This paper, thus, restricts its scope to summarizing the details on food group consumption. Among the food groups also information on oils and fats was not used to compute the dietary diversity given their strong association with obesity. The analysis is based on two rounds of cross-sectional household survey data which does not allow application of analytical tools to infer causality of the observed associations between maternal and child dietary diversity. Accordingly, the conclusions developed here refer primarily to the associative nature of mother-child diets and argues that improvements in maternal diets and asking mothers to feed various types of food consumed within the household can be helpful to enhance child dietary diversity. Also, the study design does not consider a control area which prevents us from comparing the association between maternal and child dietary diversity in intervention area which received the strengthening activities as compared to control areas where Village Health, Nutrition and Sanitation Day are organized on regular basis. Finally, the survey focused mostly on the households (mother-child dyads) whereas further research can attribute such improvements with specific components of ICDS systems strengthening.

The observed association and significant improvements in the association between maternal and child dietary diversity is consistent with experience in other low-income settings as diverse as Vietnam, Bangladesh and Ethiopia [16, 17, 23]. Policymaking can harness this potential to improve and strengthen the association across low-income communities to enhance dietary diversity among children. Project spotlight is an example that demonstrates the effectiveness of local interventions as it offers greater focus and targeting potential of resources to improve system capacities to address such perennial but addressable concern of maternal and child undernutrition. Project Spotlight, however, is not unique in such efforts and there is increasing evidence emerging from other geographies (such as Nepal and Uttar Pradesh in India) that interventions on dietary counseling and behavior change can trigger improvements in dietary diversity among children [24, 25].

The relationship between maternal and child dietary diversity in the study districts also has important implications for improving anthropometric outcomes in the region. The National Family Health Survey (2019–20) shows that 37% children (age 0–5 years) in Chandrapur district are stunted, 39% are wasted and 47% are underweight (IIPS 2020). In Gadchiroli, 36% children are stunted, 30% are wasted and 35% are underweight. While in Chandrapur, the anthropometric indicators have worsened since 2015–16 but Gadchiroli shows a mixed picture. Moreover, in both the districts one out of every four women (15–49 years) is underweight and has a low body mass index (BMI below 18.5 kg/m^2^). Harnessing the association between maternal and child dietary diversity offers the potential to improve child dietary diversity and intake can significantly influence nutritional status of children. Moreover, the evidence that mothers have higher dietary diversity than children allows expanding the scope of food group intake among children [17]. A focus on such opportunities in the first 1000 days window can be critical to stem the deterioration in the anthropometric status of children that is widely observed in the Indian context [26–29]. In this regard, it is also necessary that the dietary diversity in consumption is accompanied with adequate nutritive volume and make up to improve the association between caloric and anthropometric indicators among children [30].

The policy discussions on dietary diversity among women and children should not have a lopsided focus on anthropometric indicators. Food is the direct and most comprehensible means for improving nutritional status of women and children. Such indicators should be directly embedded within the monitoring framework of various policies and programs at all levels of administration. The Global Panel on Agriculture and Food Systems for Nutrition also calls for prioritizing strategies related to diet quality and nutrition in national plans with clear implementation mechanism and devoted leadership at all levels [31]. The Global Panel also cautions that poor diets and nutrition can lead to long-term disadvantages for development. For instance, child stunting is identified as an elementary pathway through which individuals can never attain their full potential and consequently the society also deviates from the path of developmental and prosperity. In fact, there is increasing evidence that poor dietary diversity can lead to higher prevalence of stunting [9, 32].

It is important that in the Indian context, the stagnancy in child dietary diversity receives greater policy attention. While the ICDS services aim to promote nutritional status of women and children but the strategies for promoting and monitoring dietary diversity needs to be more clearly outlined. For instance, some states in India such as Andhra Pradesh, Karnataka, Telangana, Gujarat and Maharashtra have launched hot cooked meal schemes (One Full Meal) to promote maternal dietary diversity and nutritional intake [33]. These schemes are found to have considerable impact on the maternal nutrition [34]. The low-income communities can benefit from such initiatives as they are more regular in utilization of ICDS services during the first 1000 days and beyond [23]. Besides, an excessive focus on monotonous design cereal-centric supplementary nutrition can affect coverage of these services and also has no significant impact among younger children [35–37].

Counseling strategies for maternal and child nutrition should emphasize on intake from food groups such as eggs and flesh foods as well as fruits and vegetables [17]. Higher number of food group consumption among mothers also increases the chances of intake from these food groups among children. Our analysis finds that such association is more robust in cases where mothers are reporting consumption from 5 to 6 food groups.

International evidence also supports that dietary counselling can lead to improvements in maternal and child health [5, 38–40].

In concluding, it is therefore important to reiterate the importance of dietary intake in determining nutritional status of women and children and also to highlight the case for its inclusion in nutrition policy reviews and assessments. Lopsided focus on anthropometric indicators have perhaps undermined the relevance of most fundamental determinant of maternal and child nutrition. However, interventions to enhance dietary diversity have to be well-designed and implemented to realize the specific impact of counselling on dietary diversity and translate this in terms of anthropometric improvements. Further, research on content and quality of counselling services in the context of maternal and child diets can provide more insights on strengthening such interventions.

## Supporting information

S1 Fig Dietary intake among mothers across food groups by maternal dietary diversity status, Gadchiroli and Chandrapur, 2019 and 2021. (DOCX)

S1 Table Background characteristics of study sample, Gadchiroli and Chandrapur, 2019 and 2021. (DOCX)

S2 Table Consumption of food items by children (6–23 months), Gadchiroli and Chandrapur, 2019 and 2021. (DOCX)

S3 Table Consumption of food items based on maternal and child dietary status, Gadchiroli and Chandrapur, 2019 and 2021. (DOCX)

S4 Table Logistic regression based odds ratio and AUC coefficient for the association between maternal and child dietary diversity, Gadchiroli and Chandrapur, 2019 and 2021. (DOCX)

S5 Table Logistic regression based odds ratio for the association between maternal and child dietary diversity and role of Project Spotlight, Gadchiroli and Chandrapur, 2019 and 2021. (DOCX)
