# Supplementary material for: Can Anganwadi services strengthening improve the association between maternal and child dietary diversity? Evidence from Project Spotlight implemented in tribal dominated Gadchiroli and Chandrapur districts of Maharashtra, India
Source: PLoS One. 2022 Mar 3;17(3):e0264567. doi: 10.1371/journal.pone.0264567 (PMC8893689; doi:10.1371/journal.pone.0264567)
Supplement: S1 Table — (DOCX) [file pone.0264567.s002.docx]

| Background characteristics | 2019 | | 2021 | |
| --- | --- | --- | --- | --- |
|  | N | % | N | % |
| Maternal education |  |  |  |  |
| Up to Primary | 126 | 40.3 | 139 | 36.7 |
| Above Primary | 187 | 59.7 | 240 | 63.3 |
| Maternal age |  |  |  |  |
| 15-24 years | 151 | 48.2 | 176 | 46.6 |
| 25-29 years | 128 | 40.9 | 166 | 43.9 |
| 30 years and above | 34 | 10.9 | 36 | 9.5 |
| Social group |  |  |  |  |
| Scheduled castes | 37 | 11.9 | 48 | 12.8 |
| Scheduled tribes | 152 | 48.7 | 178 | 47.5 |
| Other backward classes | 80 | 25.6 | 103 | 27.5 |
| Others | 43 | 13.8 | 46 | 12.3 |
| Sex of the child |  |  |  |  |
| Female | 157 | 50.2 | 187 | 51.8 |
| Male | 156 | 49.8 | 174 | 48.2 |
| Low birthweight of child |  |  |  |  |
| No | 218 | 69.6 | 249 | 65.7 |
| Yes | 95 | 30.4 | 130 | 34.3 |
| Self-reported economic status |  |  |  |  |
| Poor | 166 | 53.9 | 159 | 42.6 |
| Middle class or rich | 142 | 46.1 | 214 | 57.4 |
| Maternal dietary diversity |  |  |  |  |
| No | 252 | 80.5 | 238 | 62.8 |
| Yes | 61 | 19.5 | 141 | 37.2 |
