# Supplementary material for: Can Anganwadi services strengthening improve the association between maternal and child dietary diversity? Evidence from Project Spotlight implemented in tribal dominated Gadchiroli and Chandrapur districts of Maharashtra, India
Source: PLoS One. 2022 Mar 3;17(3):e0264567. doi: 10.1371/journal.pone.0264567 (PMC8893689; doi:10.1371/journal.pone.0264567)
Supplement: S2 Table — (DOCX) [file pone.0264567.s003.docx]

| Food items | 2019 | | 2021 | |
| --- | --- | --- | --- | --- |
|  | N | % | N | % |
| Any food made from beans, lentils, peas, nuts or seeds | 65 | 20.8 | 85 | 22.4 |
| Any green leafy vegetables | 96 | 30.7 | 176 | 46.4 |
| Any meat, chicken etc. | 13 | 4.2 | 34 | 9.0 |
| Any oil, fats, or butter or foods made of these | 16 | 5.1 | 23 | 6.1 |
| Any other fruits | 83 | 26.5 | 108 | 28.5 |
| Any other vegetable | 106 | 33.9 | 128 | 33.8 |
| Any sugary products such as chocolates, sweets, candies, pastries, cakes or biscuits | 183 | 58.5 | 213 | 56.2 |
| Carrots, pumpkin, sweet potato that are yellow or orange | 18 | 5.8 | 55 | 14.5 |
| Eggs | 105 | 33.5 | 211 | 55.7 |
| Fresh fish, shell fish, prawns etc. | 19 | 6.1 | 35 | 9.2 |
| Infant formula such as nestle or other local brands | 120 | 38.3 | 85 | 22.4 |
| Milk (any form of milk viz. tinned, powdered, fresh) | 164 | 52.4 | 160 | 42.2 |
| Porridge, bread, rice, roti, or things made of cereals (grains) | 273 | 87.2 | 314 | 82.8 |
| Potato, arabi, or any other root vegetable | 79 | 25.2 | 122 | 32.2 |
| Ripe mangoes, ripe papaya, watermelon, musk melon | 15 | 4.8 | 137 | 36.1 |
| Yogurt or any other milk product | 26 | 8.3 | 78 | 20.6 |
