# Supplementary material for: Can Anganwadi services strengthening improve the association between maternal and child dietary diversity? Evidence from Project Spotlight implemented in tribal dominated Gadchiroli and Chandrapur districts of Maharashtra, India
Source: PLoS One. 2022 Mar 3;17(3):e0264567. doi: 10.1371/journal.pone.0264567 (PMC8893689; doi:10.1371/journal.pone.0264567)
Supplement: S3 Table — (DOCX) [file pone.0264567.s004.docx]

| Food groups | 2019 | | | | 2021 | | | |
| --- | --- | --- | --- | --- | --- | --- | --- | --- |
|  | Both not diversified | Child Diet not diversified & mother diet diversified | Child Diet diversified & mother diet not diversified | Both diversified | Both not diversified | Child Diet not diversified & mother diet diversified | Child Diet diversified & mother diet not diversified | Both diversified |
| Grains, white roots and tubers | 82.8 | 80 | 98.8 | 90.3 | 62.5 | 88.9 | 97.3 | 97.9 |
| Pulses, nuts and seeds | 5.3 | 16.7 | 43.4 | 48.4 | 5.5 | 2.2 | 33.6 | 41.7 |
| Dairy | 58 | 63.3 | 86.7 | 83.9 | 40.6 | 37.8 | 82.7 | 77.1 |
| Meat, poultry, fish | 4.1 | 10 | 14.5 | 22.6 | 0.8 | 4.4 | 20.9 | 26 |
| Eggs | 21.3 | 10 | 56.6 | 61.3 | 31.3 | 28.9 | 80 | 72.9 |
| Vitamin A rich fruits and vegetables | 8.9 | 20 | 69.9 | 83.9 | 24.2 | 44.4 | 90 | 88.5 |
| Other fruits and vegetables | 31.4 | 33.3 | 77.1 | 80.6 | 17.2 | 33.3 | 78.2 | 81.3 |
