# Supplementary material for: Can Anganwadi services strengthening improve the association between maternal and child dietary diversity? Evidence from Project Spotlight implemented in tribal dominated Gadchiroli and Chandrapur districts of Maharashtra, India
Source: PLoS One. 2022 Mar 3;17(3):e0264567. doi: 10.1371/journal.pone.0264567 (PMC8893689; doi:10.1371/journal.pone.0264567)
Supplement: S4 Table — (DOCX) [file pone.0264567.s005.docx]

| Background variables | Simple Logistic | | | | Multilevel Logistic | | | |
| --- | --- | --- | --- | --- | --- | --- | --- | --- |
|  | 2019 | 2019 | 2021 | 2021 | 2019 | 2019 | 2021 | 2021 |
| Maternal dietary diversity |  |  |  |  |  |  |  |  |
| No | 1 | 1 | 1 | 1 | 1 | 1 | 1 | 1 |
| Yes | 2.10* | 2.03* | 2.48*** | 2.26*** | 2.10* | 2.01* | 2.48*** | 2.26*** |
|  | [1.19,3.71] | [1.10,3.77] | [1.60,3.84] | [1.40,3.65] | [1.19,3.71] | [1.08,3.74] | [1.60,3.84] | [1.40,3.65] |
| Maternal education |  |  |  |  |  |  |  |  |
| Upto Primary |  | 1 |  | 1 |  | 1 |  | 1 |
| Above Primary |  | 0.89 |  | 1.47 |  | 0.89 |  | 1.47 |
|  |  | [0.53,1.49] |  | [0.90,2.40] |  | [0.53,1.49] |  | [0.90,2.40] |
| Maternal age |  |  |  |  |  |  |  |  |
| 15-24 years |  | 1 |  | 1 |  | 1 |  | 1 |
| 25-29 years |  | 1.26 |  | 0.93 |  | 1.29 |  | 0.93 |
|  |  | [0.74,2.13] |  | [0.58,1.48] |  | [0.75,2.21] |  | [0.58,1.48] |
| 30 years and above |  | 1.82 |  | 1 |  | 1.9 |  | 1 |
|  |  | [0.83,4.02] |  | [0.45,2.21] |  | [0.85,4.25] |  | [0.45,2.21] |
| Social group |  |  |  |  |  |  |  |  |
| SC |  | 1 |  | 1 |  | 1 |  | 1 |
| ST |  | 1.46 |  | 1.16 |  | 1.64 |  | 1.16 |
|  |  | [0.65,3.27] |  | [0.58,2.32] |  | [0.66,4.09] |  | [0.58,2.32] |
| OBC |  | 1.73 |  | 1.8 |  | 1.77 |  | 1.8 |
|  |  | [0.72,4.16] |  | [0.84,3.87] |  | [0.73,4.28] |  | [0.84,3.87] |
| Other |  | 1.22 |  | 1.53 |  | 1.25 |  | 1.53 |
|  |  | [0.46,3.22] |  | [0.63,3.69] |  | [0.47,3.33] |  | [0.63,3.69] |
| Sex of the child |  |  |  |  |  |  |  |  |
| Female |  | 1 |  | 1 |  | 1 |  | 1 |
| Male |  | 0.62 |  | 0.93 |  | 0.62 |  | 0.93 |
|  |  | [0.38,1.01] |  | [0.60,1.45] |  | [0.38,1.01] |  | [0.60,1.45] |
| Low birthweight of child |  |  |  |  |  |  |  |  |
| No |  | 1 |  | 1 |  | 1 |  | 1 |
| Yes |  | 0.89 |  | 0.56* |  | 0.9 |  | 0.56* |
|  |  | [0.52,1.51] |  | [0.35,0.90] |  | [0.53,1.53] |  | [0.35,0.90] |
| Self-reported economic status |  |  |  |  |  |  |  |  |
| Poor |  | 1 |  | 1 |  | 1 |  | 1 |
| Middle-Rich |  | 1.4 |  | 0.53* |  | 1.47 |  | 0.53* |
|  |  | [0.84,2.31] |  | [0.33,0.87] |  | [0.86,2.50] |  | [0.33,0.87] |
| Observations | 313 | 307 | 379 | 351 | 313 | 307 | 379 | 351 |
| AUC | 0.56 | 0.65 | 0.60 | 0.67 | 0.56 | 0.66 | 0.60 | 0.67 |
| 95% CI | [0.51,0.61] | [0.59,0.71] | [0.56,0.65] | [0.62,0.73] | [0.51,0.61] | [0.6,0.72] | [0.56,0.65] | [0.62,0.73] |
