# Supplementary material for: Can Anganwadi services strengthening improve the association between maternal and child dietary diversity? Evidence from Project Spotlight implemented in tribal dominated Gadchiroli and Chandrapur districts of Maharashtra, India
Source: PLoS One. 2022 Mar 3;17(3):e0264567. doi: 10.1371/journal.pone.0264567 (PMC8893689; doi:10.1371/journal.pone.0264567)
Supplement: S5 Table — (DOCX) [file pone.0264567.s006.docx]

| Background variables | Chandrapur | | | Gadchiroli | | |
| --- | --- | --- | --- | --- | --- | --- |
|  | Model-1 | Model-2 | Model-3 | Model-1 | Model-2 | Model-3 |
| Maternal dietary diversity | |  |  |  |  |  |
| No | 1 | 1 |  | 1 | 1 |  |
| Yes | 2.02** | 1.77* |  | 2.79*** | 2.53** |  |
|  | [1.26,3.23] | [1.06,2.96] |  | [1.67,4.65] | [1.43,4.48] |  |
| Year |  |  |  |  |  |  |
| pid=0 | 1 | 1 |  | 1 | 1 |  |
| pid=1 | 1.88** | 1.99** |  | 1.74* | 1.76* |  |
|  | [1.23,2.87] | [1.26,3.16] |  | [1.09,2.79] | [1.07,2.92] |  |
| MDD Status x Year |  |  |  |  |  |  |
| No # pid=0 |  |  | 1 |  |  | 1 |
| No # pid=1 |  |  | 2.20** |  |  | 1.54 |
|  |  |  | [1.28,3.77] |  |  | [0.86,2.73] |
| Yes # pid=0 |  |  | 2.19 |  |  | 1.76 |
|  |  |  | [0.99,4.85] |  |  | [0.69,4.50] |
| Yes # pid=1 |  |  | 3.38*** |  |  | 4.76*** |
|  |  |  | [1.71,6.65] |  |  | [2.39,9.47] |
|  |  |  |  |  |  |  |
| Upto Primary |  | 1 | 1 |  | 1 | 1 |
| Above Primary |  | 1.43 | 1.44 |  | 0.84 | 0.87 |
|  |  | [0.88,2.33] | [0.88,2.34] |  | [0.49,1.44] | [0.50,1.49] |
| Maternal age |  |  |  |  |  |  |
| 15-24 years |  | 1 | 1 |  | 1 | 1 |
| 25-29 years |  | 1.23 | 1.21 |  | 0.92 | 0.91 |
|  |  | [0.77,1.96] | [0.76,1.94] |  | [0.54,1.57] | [0.53,1.55] |
| 30 years and above |  | 1.2 | 1.22 |  | 1.61 | 1.61 |
|  |  | [0.45,3.18] | [0.46,3.24] |  | [0.78,3.33] | [0.78,3.32] |
| Social group |  |  |  |  |  |  |
| SC |  | 1 | 1 |  | 1 | 1 |
| ST |  | 1.99 | 1.99 |  | 0.72 | 0.71 |
|  |  | [0.98,4.04] | [0.98,4.05] |  | [0.30,1.72] | [0.30,1.71] |
| OBC |  | 1.65 | 1.65 |  | 1.29 | 1.25 |
|  |  | [0.83,3.28] | [0.83,3.28] |  | [0.43,3.84] | [0.42,3.74] |
| Other |  | 1.91 | 1.88 |  | 0.56 | 0.57 |
|  |  | [0.89,4.10] | [0.87,4.04] |  | [0.16,1.99] | [0.16,2.02] |
| Sex of the child |  |  |  |  |  |  |
| Female |  | 1 | 1 |  | 1 | 1 |
| Male |  | 0.74 | 0.75 |  | 0.76 | 0.76 |
|  |  | [0.47,1.16] | [0.48,1.17] |  | [0.46,1.23] | [0.47,1.24] |
| Low birth weight of child | |  |  |  |  |  |
| No |  | 1 | 1 |  | 1 | 1 |
| Yes |  | 0.84 | 0.84 |  | 0.66 | 0.66 |
|  |  | [0.51,1.39] | [0.51,1.39] |  | [0.40,1.08] | [0.40,1.09] |
| Self-reported economic status | |  |  |  |  |  |
| Poor |  | 1 | 1 |  | 1 | 1 |
| Middle-Rich |  | 0.82 | 0.82 |  | 1.04 | 1.06 |
|  |  | [0.51,1.32] | [0.51,1.31] |  | [0.60,1.77] | [0.62,1.82] |
| Observations | 367 | 346 | 346 | 325 | 312 | 312 |
