# Supplementary material for: Can Anganwadi services strengthening improve the association between maternal and child dietary diversity? Evidence from Project Spotlight implemented in tribal dominated Gadchiroli and Chandrapur districts of Maharashtra, India
Source: PLoS One. 2022 Mar 3;17(3):e0264567. doi: 10.1371/journal.pone.0264567 (PMC8893689; doi:10.1371/journal.pone.0264567)
Supplement: Supplementary file 6 [file pone.0264567.s001.docx]

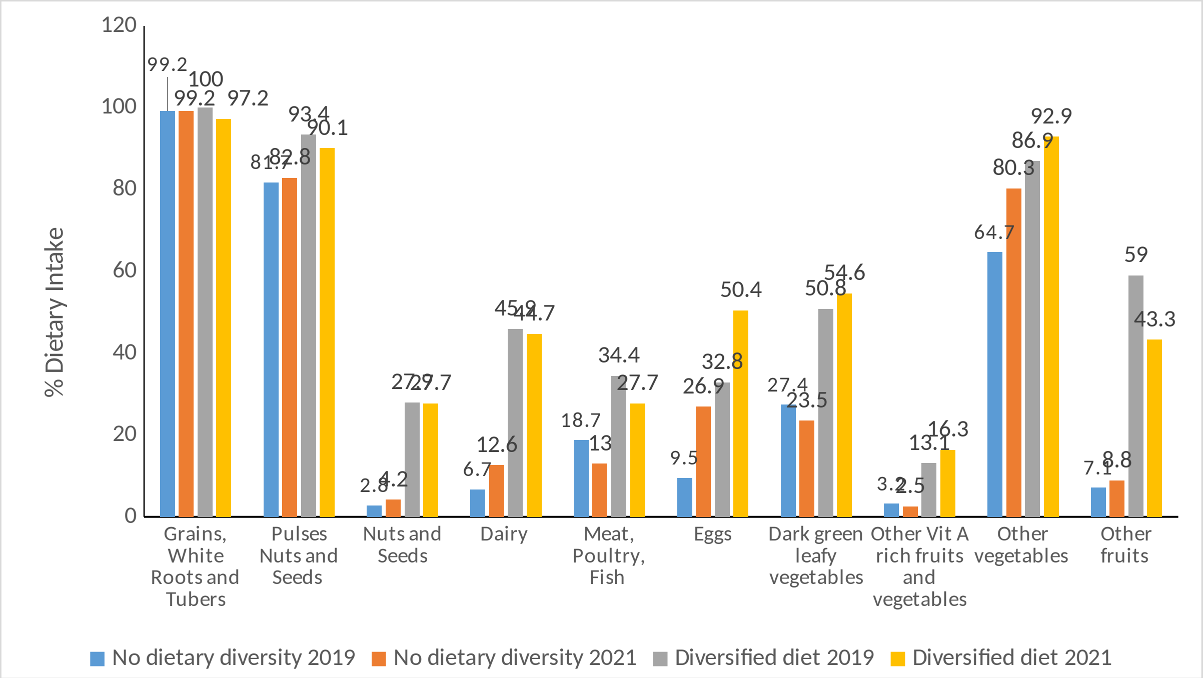

% Dietary Intake
120
100
80
60
40
20
0
99.2
100
99.2
97.2
81.7
82.8
93.4
90.1
2.8
4.2
27.9
27.7
6.7
12.6
45.9
44.7
18.7
13
34.4
27.7
9.5
26.9
32.8
50.4
27.4
23.5
50.8
54.6
3.2
2.5
13.1
16.3
64.7
80.3
86.9
92.9
7.1
8.8
59
43.3
Grains, White Roots and Tubers
Pulses Nuts and Seeds
Nuts and Seeds
Dairy
Meat, Poultry, Fish
Eggs
Dark green leafy vegetables
Other Vit A rich fruits and vegetables
Other vegetables
Other fruits
No dietary diversity 2019
No dietary diversity 2021
Diversified diet 2019
Diversified diet 2021
